# Shorter and Longer Antibiotic Durations for Respiratory Infections: To Fight Antimicrobial Resistance—A Retrospective Cross-Sectional Study in a Secondary Care Setting in the UK

**DOI:** 10.3390/ph17030339

**Published:** 2024-03-06

**Authors:** Rasha Abdelsalam Elshenawy, Nkiruka Umaru, Zoe Aslanpour

**Affiliations:** Department of Clinical Pharmacy and Pharmaceutical Sciences, School of Life and Medical Sciences, University of Hertfordshire, Hatfield AL10 9AB, UK

**Keywords:** antimicrobial resistance, antibiotic duration, antibiotics, respiratory tract infections, COVID-19 pandemic, antimicrobial stewardship, antibiotic prescribing

## Abstract

As antimicrobial resistance (AMR) escalates globally, examining antibiotic treatment durations for respiratory infections becomes increasingly pertinent, especially in the context of the COVID-19 pandemic. In a UK secondary care setting, this retrospective study was carried out to assess the appropriateness of antibiotic treatment durations—shorter (≤5 days) versus longer (6–7 days and >8 days)—for respiratory tract infections (RTIs) in 640 adults across 2019 and 2020, in accordance with local antimicrobial guidelines. The analysis employed these guidelines and clinical evidence to examine the effectiveness and suitability of antibiotic prescribing practices. This study considered the ‘Shorter Is Better’ approach, noting an increased rate of patient discharges associated with shorter antibiotic regimens (≤5 days). It further demonstrates that shorter treatments are as effective as longer ones for conditions such as COPD exacerbation, COVID-19 pneumonia, and hospital-acquired pneumonia (HAP), except in cases of community-acquired pneumonia (CAP) and unspecified diagnoses. Nevertheless, this study raises concerns over an observed increase in mortality risk with shorter treatment durations. Although these mortality differences were not statistically significant and might have been influenced by the COVID-19 pandemic, the need for extended research with a larger sample size is highlighted to confirm these findings. This study also emphasises the critical need for accurate and specific diagnoses and considering risk assessments at admission, advocating for tailored, evidence-based antibiotic prescribing to ensure patient safety. It contributes to antimicrobial stewardship efforts by reinforcing the importance of adapting antibiotic use to current healthcare challenges and promoting a global commitment to fight antimicrobial resistance. This approach is crucial for enhancing patient outcomes and saving lives on a global scale.

## 1. Introduction

In the face of rising antimicrobial resistance (AMR), the global health landscape is rapidly changing [1]. This resistance threatens the efficacy of conventional treatments such as antibiotics, chemotherapy, and various pharmaceuticals [2]. Recognising the severity of AMR, the World Health Organisation (WHO) has classified it among the ten global threats to health worldwide, calling for the prudent use of antibiotics [3]. To address this crisis, the development of novel strategies and the reinforcement of existing treatments are at the forefront of scientific research [4]. With infectious diseases becoming more prevalent and pathogens increasingly outpacing current treatments, the necessity for diverse approaches in combating AMR has never been more crucial [5,6].

In response to this crisis, antimicrobial stewardship (AMS) initiatives have become crucial, promoting the responsible use of antibiotics to mitigate the risks associated with AMR [7,8]. The importance of AMS programs is highlighted by the rapid spread of AMR, which complicates the management of infectious diseases [9,10]. This increase in resistance leads to longer hospital stays, spiralling healthcare costs, and a rise in mortality rates, highlighting the urgent need for effective AMS measures [10,11]. Implementing AMS strategies, such as ‘IV-to-Oral Switch’, ‘Discontinuing Antibiotics’, and ‘De-escalation’, is essential to ensure the effective enactment of AMS [12]. The development of new AMS strategies is critical, especially in the context of the COVID-19 pandemic, which influenced antibiotic prescribing practices and exacerbated the misuse of antibiotics [13,14]. Integrating this new AMS strategy, refining existing practices, and enacting robust preventive measures can enhance our defences against infectious diseases and more effectively tackle the escalating issue of AMR [13,15].

Recent evidence suggests that short-course antibiotic therapy can be as effective as longer courses, prompting a re-evaluation of prescribing practices to mitigate the emergence of AMR [13,16,17]. This shift towards shorter courses is supported by studies demonstrating comparable outcomes between short and long therapies, marking a significant change in clinical practice standards [18]. In the UK, efforts to reduce antibiotic resistance include addressing antibiotic over-prescribing, where substantial evidence indicates that reducing antibiotic use could lower or stabilise resistance levels [19]. This involves starting treatments only when necessary, selecting appropriate drugs, and avoiding unnecessarily long treatment durations. However, less attention has been paid to minimising prolonged treatment durations as a strategy to control antibiotic overuse in primary care [6,20]. Additionally, the side effects associated with antibiotic use, such as diarrhoea, rash, and candidiasis, highlight the importance of minimising treatment duration to reduce the risk of adverse outcomes, including Clostridium difficile infections [21]. Recent guidelines and studies advocate for shorter antibiotic courses for common infections, suggesting that such practices can effectively clear infections while minimising the selection and spread of resistant bacteria [22]. The principle of administering the minimum effective duration of antibiotic therapy to reduce AMR risk and drug toxicity is a cornerstone of AMS, with evidence from randomised controlled trials supporting short courses for lower respiratory tract infections [18]. However, the optimal duration of antibiotic therapy for pleural infection remains unclear, with limited high-quality evidence available [23].

This study aims to compare the effectiveness and appropriateness of shorter versus longer antibiotic treatment durations for respiratory tract infections in adults during 2019 and 2020. It categorises the antibiotic treatments into three durations: shorter duration (5 days or fewer), longer duration (6–7 days), and over 8 days. Additionally, this study explores factors that might justify a more prolonged course of antibiotic therapy. This research contributes to bridging gaps in the existing literature by offering a comparative analysis of antibiotic treatment durations at a secondary care setting, one National Health Service (NHS) Foundation Trust in the UK, in 2019 (prior to the COVID-19 pandemic) and in 2020 during the pandemic. This NHS Foundation Trust is a secondary care provider serving around 700,000 people in the East of England region of the UK.

## 2. Results

### 2.1. Categorising Antibiotic Treatment Durations: Shorter and Longer

The flow chart presents the categorisation of 640 patients based on antibiotic treatment duration and respiratory diagnoses, with durations segmented into ‘Shorter Duration’ (≤5 days) and ‘Longer Duration’ (6–7 days and >8 days). This categorisation is derived from local antimicrobial guidelines, a review of the literature, and the clinical relevance of antibiotic duration practices. Of 640 patients in total, admitted in 2019 and 2020, 463 patients received antibiotics for ≤5 days, 109 for 6–7 days, and 68 for periods exceeding 8 days. Based on the information extracted from patients’ medical records, primary diagnoses were categorised by specific respiratory infections. These include community-acquired pneumonia (CAP), hospital-acquired pneumonia (HAP), ventilator-associated pneumonia (VAP), chronic obstructive pulmonary disease (COPD) infective exacerbation without pneumonia, and COVID-19 pneumonia. Alongside these, indeterminate diagnoses such as upper respiratory tract infections (URTIs), lower respiratory tract infections (LRTIs), or pneumonia were grouped under the ‘Unspecified’ category for respiratory tract infections (RTIs).

**Figure 1:**
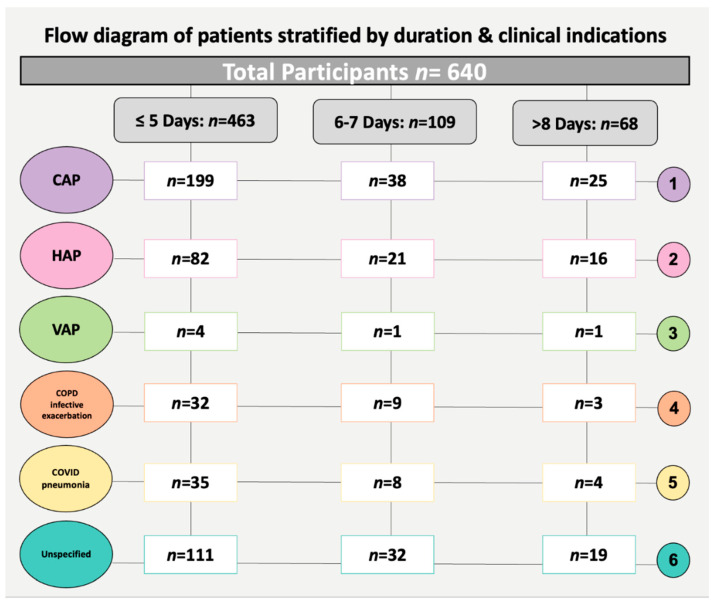
Flow diagram for extracting a representative sample of 640 patient medical records from 2019 to 2020, stratified by duration and clinical indications. CAP, community-acquired pneumonia; HAP, hospital-acquired pneumonia; VAP, ventilator-associated pneumonia; COPD, chronic obstructive pulmonary disease; COVID-19, coronavirus.

In the CAP category, treatment durations were as follows: 199 patients received antibiotics for ≤5 days, 38 for 6–7 days, and 25 for periods exceeding 8 days, illustrating the variation in treatment lengths for different respiratory conditions. For HAP, 82 patients were treated for ≤5 days, 21 for 6–7 days, and 16 for more than 8 days. Regarding COPD exacerbations and COVID-19 pneumonia, 32 and 35 patients, respectively, were treated for a shorter duration of ≤5 days. For patients with ‘Unspecified’ RTIs, 111 received a shorter treatment course of ≤5 days (Figure 1).

### 2.2. Clinical and Demographic Characteristics

Table 1 represents the patient characteristics and clinical features of 640 patients hospitalised prior to and during the COVID-19 pandemic across 2019 and 2020, categorised by antibiotic treatment duration: ≤5 days, 6–7 days, and >8 days. Regarding age, the median values are relatively consistent across the categories, recorded as 79, 80, and 79.5 for the ≤5-day, 6–7-day, and >8-day groups, respectively. No significant differences were observed in white blood cells (WBCs) or C-reactive protein (CRP), with *p*-values of 0.3 and 0.7, accordingly. Length of stay (LOS) demonstrated significant differences, with longer stays correlating with antibiotic use exceeding 8 days, as indicated by a *p*-value of <0.01.

In Table 2, it was found in 2019 and 2020, most of the patients, 237 (73.6%), had shorter appropriate antibiotic courses of ≤5 days in 2019 and 226 (71.1%) in 2020. No significant difference was observed in antibiotic duration based on year, gender, outcomes (discharge or death), allergies, or clinical characteristics, such as hypertension (HTN), heart failure (HF), diabetes mellitus (DM), and asthma. Most of the study population, 384 (82.9%), were discharged with shorter antibiotic courses of ≤5 days, with no significant difference between the three categories. For the patient outcome ‘died,’ it was found that 79 died in the ≤5-day antibiotic duration category, representing a mortality rate of 17.1%. The mortality rate decreased to 11% for the 6–7-day duration and further to 10.3% for durations > 8 days. Upon comparing chest X-ray results, it was revealed that within the ≤5-day antibiotic treatment duration, pneumonia was present in 66 cases (14.3%), and no pneumonia in 107 cases (23.1%), with no significant statistical difference observed across the categories (*p*-value = 0.3).

### 2.3. Most Frequently Prescribed Antibiotics for Respiratory Infections

Table 3 outlines the most commonly prescribed antibiotics for patients with RTIs in a UK secondary care setting over two years, 2019 and 2020. The antibiotics are categorised by the duration of their administration: ≤5 days, 6–7 days, and >8 days. Amoxicillin/clavulanic acid was the most frequently prescribed antibiotic, with 283 (61.1%) prescriptions for ≤5 days. Additionally, levofloxacin accounted for 50 (10.8%) of its use within the shorter-duration category of ≤5 days. There was no significant difference in the durations for most of the prescribed antibiotics, except for metronidazole and piperacillin/tazobactam, which had *p*-values of 0.01 and 0.007, respectively.

### 2.4. Shorter versus Longer Antibiotic Courses in Respiratory Infections

A key finding of this study was the assessment of the appropriateness of initial or empirical antibiotic prescribing in accordance with local guidelines. Appropriate prescribing was evaluated by comparing the antibiotics prescribed to patients admitted in 2019, prior to the pandemic, and in 2020, during the pandemic, against hospital or local antimicrobial guidelines (BSAC Stewardship, 2018). This study focused on analysing the differences between shorter and longer courses of appropriate antibiotic therapy for various RTIs. For instance, with CAP, local guidelines recommended an antibiotic treatment duration ranging from 5 days (shorter duration) to longer durations of 6–7 days and >8 days. Similarly, in cases of COPD infective exacerbation, a shorter antibiotic course of ≤5 days was assessed against longer durations of 6–7 days and >8 days.

To examine the appropriateness of initial or empirical antibiotic prescribing in accordance with local guidelines, appropriate prescribing was evaluated by comparing the antibiotics prescribed to patients admitted in 2019, prior to the pandemic, and in 2020, during the pandemic, against the hospital or local antimicrobial guidelines for the RTI conditions, such as HAP, VAP, COPD infective exacerbation, and COVID-19 pneumonia. Table 4 presents a comparison of appropriate antibiotic prescribing in the shorter versus longer durations of antibiotic treatment in the study population. A ‘Shorter Duration’ of ≤5 days was shown to be as effective as ‘Longer Durations’ of 6–7 days and >8 days. There was no significant difference in the appropriateness of shorter versus longer antibiotic durations among the three RTI categories, with the exceptions of CAP, which showed a *p*-value of 0.02, and ‘Unspecified’ RTIs, which had a *p*-value of 0.07. 

Furthermore, the majority of patients were appropriately prescribed antibiotics for shorter durations of ≤5 days, representing 164 (35.4%) of the cases. No significant difference was observed in the appropriateness of shorter versus longer durations of antibiotic prescribing across the three categories in the overall study population. COVID-19 and VAP cases were fewer in number, with varied appropriateness across durations. The data presented in the table suggest a move towards prescribing shorter courses of antibiotics, a change that aligns with current efforts to fight antimicrobial resistance. This shift becomes particularly notable in the management of patients with COVID-19.

## 3. Discussion

This study provides valuable insights into the challenges and adjustments in antimicrobial stewardship during an unprecedented global health crisis. This, in turn, informs future strategies and policy modifications in combating AMR and managing global health emergencies [24]. The historical 7 days for antibiotic therapy has long been challenged, especially for pneumonia treatments. Studies have revealed that short-course treatments (3–5 days) are just as effective for community-acquired pneumonia, and ≤8 days are sufficient for nosocomial pneumonia, compared to the conventional 7–10 or 10–15 days [25]. This not only counters the misconception that prolonged treatment prevents resistance but highlights that longer treatments may increase resistance emergence [26]. The Antibiotic Mantra, ‘Shorter Is Better’, advocates for therapy durations tailored to the patient’s response, shifting from the outdated practice of fixed, extended courses to a more evidence-based, patient-specific approach [27]. In this study, for patients with CAP, 199 patients were treated for shorter durations of ≤5 days, 38 for 6–7 days, and 25 for more than 8 days. A statistically significant difference was observed across the three duration categories, with CAP showing a *p*-value of 0.02. However, in the case of hospital-acquired pneumonia (HAP), there was no significant difference between the three categories, with a *p*-value of 0.7.

In 2018, Public Health Ontario (PHO) launched the ‘Shorter is Smarter’ initiative, highlighting the critical need to reduce the duration of antibiotic therapy in long-term care settings. This initiative sheds light on the concept of selective pressure, where antibiotic use can eliminate susceptible bacteria and allow resistant strains to multiply [28,29]. Advocating for shorter courses of antibiotics demonstrates their effectiveness compared to longer durations for treating conditions like pneumonia. These shorter courses, ranging from 5 to 6 days as opposed to the traditional 7 to 14 days, aim to decrease resistance and side effects [30,31]. Supported by studies on common infections among both hospitalised and ambulatory long-term-care patients, this strategy encourages minimising antibiotic use to reduce harm [32,33,34,35,36].

In confronting antibiotic resistance, prescribing fewer antibiotics is crucial. A 7-day treatment, a vestige of Constantine the Great’s decree, lacks evidence for modern medicine [27]. Over 45 RCTs now affirm that shorter courses are as effective for various infections, including pneumonia. For instance, 3–5-day treatments for community-acquired pneumonia and ≤8 days for nosocomial pneumonia are proven effective [37]. Each additional day of antibiotics raises adverse effects by 5%, compelling the medical community to embrace the ‘shorter is better’ approach for better outcomes and less resistance [16,38].

In 2021, a study examining short versus long antibiotic courses for treating infections revealed no difference in effectiveness but found that longer durations were linked to more hospital admissions due to complications [39]. Research on 4 million cases in England indicated that prescriptions of 8–15 days had higher risks compared to shorter treatments. The findings support the use of brief courses in combating antimicrobial resistance and suggest a shift in clinical guidelines towards shorter antibiotic durations [17]. 

Although it was found in this study that longer antibiotic durations are associated with a lower mortality rate of 10.3%, this difference is not statistically significant (*p*-value = 0.6), suggesting that duration may not influence survival rates. Shorter antibiotic treatment durations have a high discharge rate of 82.9%, indicating effectiveness. There is a significant increase in the length of stay for patients on antibiotics for more than 8 days (*p*-value < 0.01), implying that longer antibiotic treatment durations could lead to prolonged hospital stays. This, however, does not necessarily correlate with improved survival within the study population. This study observed higher mortality rates among patients who received shorter antibiotic treatments; however, these results were not statistically significant. The 2020 sample of the study, accounting for half of the patient records, suggests that the COVID-19 pandemic may have contributed to increased mortality. As of 2020, the WHO estimated at least 3 million global deaths due to COVID-19, which is significantly higher than the reported figures, indicating an undercount of direct and indirect pandemic-related fatalities [40,41]. This indicates the necessity for further research emphasising the need for a larger sample size, particularly to understand the influence of pandemics and antibiotic duration on such trends. However, this finding highlights the need for meticulous consideration in determining the duration of antibiotic therapy, guided by local or hospital guidelines and the severity of the patient’s condition. It is imperative to ensure that recommendations are supported by robust, comprehensive evidence that also considers patient safety, urging a more evidence-based approach in antibiotic management strategies and antimicrobial stewardship implementation to save patient lives. The systematic review and meta-analysis conducted in 2023 aimed to ascertain the optimal duration of antibiotic treatment for adults with CAP. The analysis of nine trials involving 2399 patients indicated that shorter courses of antibiotics, ranging from 3 to 5 days, might be non-inferior to the conventional 10-day regimen without increasing adverse outcomes. These findings imply that shorter treatments could be more efficacious and less burdensome if patients have reached clinical stability. However, the conclusions are tempered by the small number of studies and the potential for bias. Additional research is necessary, especially for patients with severe illness [42]. This study does not provide sufficient evidence to support the superiority of shorter antibiotic treatment durations over longer ones. However, shorter durations, indicated by a high discharge rate of 82.9%, suggest potential effectiveness and should be considered. Longer durations may be necessary depending on the patient’s clinical condition, especially in cases of severe illness, highlighting the need for further research.

In 2023, a study conducted in the UK evaluated antibiotic use in patients with RTIs using the WHO AWaRe classification. Notably, it observed a slight increase in the use of amoxicillin/clavulanic acid and a substantial rise in azithromycin prescriptions, highlighting shifts in prescribing trends. Despite these changes, some antibiotics displayed steady consumption rates. These findings highlight the importance of understanding antibiotic use patterns during the AMR threat. The increase in the usage of ‘Watch’-category antibiotics during the pandemic emphasises the urgency of robust AMS measures. The research confirms that incorporating the AWaRe classification in prescribing decisions is crucial for patient safety and combating antibiotic misuse. This study provides essential insights into the changing landscape of antibiotic prescribing during a global health crisis, reinforcing the necessity for ongoing AMS vigilance to effectively address AMR challenges [43,44]. This study revealed that amoxicillin/clavulanic acid was the antibiotic most commonly prescribed, accounting for 283 (61.1%) of prescriptions lasting ≤5 days. For the majority of antibiotics prescribed, the duration did not significantly vary, with the exceptions being metronidazole and piperacillin/tazobactam, which showed significant differences with *p*-values of 0.01 and 0.007, respectively. In 2023, another study in Japan showed that a shorter treatment duration (3–5 days) likely offers the best balance of effectiveness and treatment burden for managing CAP in adults who have achieved clinical stability. Nevertheless, the limited number of studies considered and the overall moderate-to-high risk of bias could affect the reliability of these findings. Additional research focusing on this shorter duration of treatment is necessary [43].

A study conducted in the USA at an academic children’s hospital from January 2017 to May 2020 evaluated antibiotic treatment durations for culture-negative sepsis in paediatric ICU patients. It revealed that a short course (≤7 days) resulted in lower mortality and shorter hospital stays compared to a long course (>7 days), with no significant difference in 30-day mortality or multidrug-resistant organism (MDRO) acquisition. These findings suggest the potential efficacy of shorter antibiotic therapies, emphasising the need for further extensive research to validate these results and inform future sepsis treatment protocols [45]. Implementing antimicrobial stewardship and focusing on antibiotic safety are crucial steps in combating antibiotic resistance, an issue highlighted by numerous studies and initiatives [46]. By adopting new AMS strategies, emphasising shorter durations of antibiotics, enhancing our current guidelines, and implementing effective disease prevention measures, we can bolster our safeguards against infections and more effectively address the rising challenge of AMR. This approach ensures the safe and judicious use of antibiotics, contributing to better health outcomes [16,29,47,48].

### Strengths and Limitations

This study offers pivotal insights into optimising antibiotic therapy durations, particularly underlining the efficacy of shorter antibiotic duration. For example, it finds that ≤5-day antibiotic courses for COPD infective exacerbation and COVID-19 pneumonia are just as effective as longer ones, challenging the traditional 7–10-day regimens and suggesting a shift towards more tailored, patient-specific approaches. Additionally, the ‘Shorter is Smarter’ initiative by Public Health Ontario and findings from multiple RCTs support the move to shorter courses, aiming to reduce antibiotic resistance and adverse effects. This discussion synthesises evidence from around the globe, including significant research from the UK, USA, and Japan, reinforcing the mantra ‘shorter is better’ for antibiotics. Such insights are pivotal for evolving AMS strategies and adapting to the nuances of managing infections during health crises, emphasising the need for ongoing research to refine antibiotic use and enhance patient care. 

However, this study faces certain limitations. The focus on an adult population, excluding individuals under 25 and children, restricts its demographic applicability. This study is limited by its retrospective observational design, which affects the validity of comparisons between short and long antibiotic treatment durations. Future randomised controlled trials (RCTs) are necessary for more robust insights due to potential biases not fully addressed in the current study. RCTs are needed to investigate whether shorter antibiotic treatments are not inferior to or even more effective than longer durations and allow for efficacy comparisons. A key limitation of this study is its potential lack of statistical power to identify significant differences among RTI antibiotic treatment duration groups, stemming from small sample sizes. Further investigations are needed to examine the relationship between antibiotic duration, mortality rates, and LOS, highlighting the importance of additional studies with larger cohorts to confirm these results.

Additionally, being a monocentric study raises concerns about the generalisability of the results. Further studies are essential. Its focus solely on RTIs limits the scope of the findings. The brief duration of the study and the evolving nature of SARS-CoV-2 could also impact the results. While the findings offer valuable insights into antibiotic use during a pivotal time of the COVID-19 pandemic in 2020, they should be interpreted with these limitations in mind, highlighting the need for continuous research to understand healthcare professionals’ antibiotic prescribing practices during pandemic conditions. Despite these promising outcomes, this research draws attention to concerning findings, such as an elevated mortality risk associated with shorter treatment durations. Although these differences in mortality rates were not statistically significant, they emphasise the necessity for a larger sample size, particularly to understand the pandemic’s influence on such trends. The results from this study have also emphasised the importance of clear and precise diagnoses and the application of severity scoring tools for guiding appropriate antibiotic usage, which is key for managing AMR. Furthermore, this study corroborates the efficacy of shorter antibiotic courses, in line with local guidelines and clinical evidence. This strategy should be integrated into emergency planning while maintaining adherence to best practices.

## 4. Materials and Methods

### 4.1. Study Design and Setting

This cross-sectional retrospective study was conducted in a secondary care setting, one NHS Foundation Trust in the East of England region of the UK. The study aimed to estimate the prevalence of appropriate antibiotic prescribing among shorter and longer durations of antibiotics in adult patients aged 25 years and above who were admitted to a secondary care setting in the UK during 2019 (prior to the pandemic) and 2020 (during the pandemic). The study was conducted from 1 August 2021 to 28 February 2023. Serving approximately 700,000 people, this secondary care provider is equipped with about 742 beds. This study’s findings were reported in line with the STROBE (Strengthening the Reporting of Observational Studies in Epidemiology) statement [49].

### 4.2. Participants

To optimise participant diversity, this investigation adopted a stratified sampling methodology for the selection of medical records. The cohort encompassed adults aged 25 years and above, including pregnant women and individuals with compromised immune systems who were admitted to the secondary care setting during the years 2019 and 2020. The focus was particularly on those administered antibiotics for RTIs, covering instances of pneumonia across both years and extending to COVID-19 in 2020. Exclusion criteria were established for individuals who had a stay of less than 48–72 h in the accident and emergency (A&E) department, those not administered antibiotics, and paediatric patients. The research protocol underwent evaluation and received input from the Citizens’ Senate, an entity championing patient care with substantial representation of the elderly demographic. 

This study has been officially registered with the ISRCTN registry. The ISRCTN registry is a primary registry related to WHO criteria and the International Committee of Medical Journal Editors (ICMJE), accepting all clinical research studies (ISRCTN 14825813) [50]. It was also registered in Octopus, the global primary research record [51].

### 4.3. Data Sources and Variables

Patient selection was based on electronic health record (EHR) entries identified by the respective ICD-10 codes for RTIs. This covered a range of conditions, encompassing both specific and indeterminate diagnoses. Specific conditions included CAP, COPD infective exacerbation, HAP, and VAP. In 2020, the selection criteria were expanded to include cases of COVID-19 pneumonia. Additionally, indeterminate diagnoses such as URTIs, LRTIs, and pneumonia were categorised as ‘Unspecific’ RTIs. The primary diagnosis of RTIs in these records played a crucial role in determining the initial or empirical antibiotic prescribed to the patients.

The sample size calculation was based on Public Health England’s estimation that 20% of all antibiotics prescribed in the UK might be inappropriate [52]. This study, a retrospective cross-sectional analysis serving the NHS Foundation Trust’s population of around 700,000 in East England, spanned two significant years: 2019 and 2020. Utilising Minitab Statistical Software Version 21.1.0 and considering the service population, a 10% margin of error, and a 95% confidence interval, the required sample size was determined. A total of 640 patient records were precisely selected to analyse antibiotic prescribing trends during the specified years.

For sampling, the systematic method was employed to consistently select patient medical record data from a larger dataset at the Trust. Initially, data from 4830 records (2755 from 2019 and 2075 from 2020) were extracted. After applying inclusion and exclusion criteria and eliminating duplicate records, the numbers were narrowed down to 1188 for 2019 and 939 for 2020. Subsequently, a random selection of 320 records for each year was conducted using Excel’s RAND function. This resulted in a total of 640 patient records. This approach streamlined the sampling process while ensuring a comprehensive representation of the patient population. 

The primary author (R.A.) extracted data from the electronic medical records of patients within the secondary care setting. These data included age, sex, allergies, indications for treatment, comorbidities, CRP levels, WBC count, chest X-ray results, and the duration of antibiotic treatment, categorised as shorter duration (≤3 days) and longer duration (6–7 days and >8 days). Additionally, the LOS and patient outcomes, whether discharged or deceased, were also recorded.

In this secondary care setting, the initiation of empirical antibiotic treatment is based on an initial, tentative diagnosis at the time of patient admission. The primary author meticulously evaluated the alignment of the chosen empirical antibiotic treatments with the local antibiotic guidelines to ascertain their appropriateness. These local guidelines serve as a gold standard, detailing the criteria for selecting empirical antibiotics, encompassing considerations for the type of infection, patient-specific factors, and local resistance patterns. The assessment process involves a thorough review of the antibiotics prescribed and examining aspects such as the type of antibiotic and prescribed duration. This review extends beyond the initial diagnosis and is dynamically adapted based on the patient’s clinical response, results from microbiological testing, and additional diagnostic procedures, such as chest X-ray findings. This method ensures that the antibiotic therapy aligns not only with the preliminary diagnosis but also remains responsive to the evolving clinical scenario and diagnostic insights, thus optimising patient care whilst adhering to antimicrobial stewardship practices.

A data extraction tool was employed to obtain the necessary data from patients’ medical records. The data extraction tool was prepared in order to obtain the necessary information from the patient’s medical records. The AMS data extraction tool was prepared, encompassing demographic information, primary diagnosis, investigations, and patient outcomes. The extraction process took approximately 45 min per patient’s medical record for the primary author to gather the required data. The primary author reviewed the literature and the UKHSA’s AMS Toolkit to develop the data extraction tool [9]. The authors discussed, recognised, and agreed to the elements within the tool. 

A pilot study was conducted to provide an initial overview of the data and to evaluate the feasibility of the data extraction tool in addressing the research questions. This pilot study aimed to provide more description of the data and examine the feasibility of the data extraction tool in answering the research questions. Data were extracted from 80 patient medical records, 40 in 2019 and 40 in 2020. In this pilot test, two independent authors separately extracted data from 1% of the sample (four patient records) to validate the data extraction tool. An agreement rate of 80% or higher was used as a measure of the tool’s validity. To assess the tool’s reliability, both authors independently extracted data from another 1% of the sample (four records). Inter-rater reliability was determined by comparing the percentage agreement in data extracted independently. Any disagreements were resolved through discussion. The result of the pilot study indicated that the data extraction tool was sufficient to address all the study objectives. Due to the small sample size of the pilot study, not all statistical analyses were applied. It was expected to include both descriptive and statistical data. It was impossible to undertake the other statistical tests. More data were required to analyse antibiotic prescribing trends during the specified years. Data generated and extracted from the pilot test were not included in the actual study analysis. 

### 4.4. Statistical Methods

Descriptive analyses were conducted. Data on categorical or binary variables were presented as numbers (*n*) and proportions (%). Initial data, including age, gender, allergies, indications, comorbidities, duration, WBC count, CRP levels, and chest X-ray results, were described using numbers (*n*) and percentages (%) and further analysed. The Chi-square test was utilised for categorical variables, and the Kruskal–Wallis test was applied to numerical variables. This study examined the appropriateness of initial or empirical antibiotic prescriptions against local guidelines. Antibiotics given to patients admitted in 2019 and 2020 were compared with the local hospital’s antimicrobial guidelines to assess the appropriateness of the prescriptions. The appropriateness of prescribed antibiotics among different indications across the three duration categories was also analysed using the Chi-square test. This study compared the appropriateness of prescribed antibiotics for RTIs in adult patients admitted in a secondary care setting across three antibiotic duration categories: shorter duration (≤5 days) and longer duration (6–7 days and >8 days). For more advanced statistical analysis, IBM SPSS Statistics version 22.0, RStudio version 2022, and R version 4.2.2 were used [53,54]. 

## 5. Conclusions

In this comprehensive study within a UK secondary care setting, 640 adult patient records from 2019 and 2020 were analysed to evaluate the effectiveness of antibiotic durations for RTIs, in the context of rising AMR and the challenges posed by the COVID-19 pandemic. This study explores the ‘Shorter Is Better’ approach, noting an increased discharge rate in the group receiving shorter antibiotic treatments (≤5 days), and demonstrating that these shorter courses are as effective as longer traditional ones for conditions such as COPD exacerbation, COVID-19 pneumonia, and HAP, with the exceptions being CAP and unspecified indications. Despite these promising results, this research highlights concerning observations, including an elevated mortality risk associated with shorter treatment durations. While these mortality rate differences were not statistically significant and could have been influenced by the pandemic, there is an emphasized need for a larger sample size to delve deeper into these findings. Crucially, this research emphasises the importance of accurate diagnoses and thorough risk assessments upon admission, advocating for customised, evidence-based antibiotic prescribing that prioritises patient safety and the safeguarding of patient lives. It aligns with local guidelines and highlights the vital role of antimicrobial stewardship in enhancing patient safety, optimising healthcare outcomes, and contributing effectively to the global effort to fight antimicrobial resistance.

## Figures and Tables

**Table 1:** Patient characteristics and clinical features by antibiotic treatment duration for 2019 and 2020 admissions (*n* = 640).

	Duration of Antibiotic Use			*p*-Value
	≤5 Days	6–7 Days	>8 Days	
	*n (*%)	*n (*%)	*n (*%)	
Age (Median, IQR)	79 (21)	80 (17)	79.5 (19)	0.8
WBCs (Median, IQR)	34.1 (123)	46 (131)	16.5 (117)	0.3
CRP (Median, IQR)	76 (206)	91 (291)	103.5 (240)	0.7
LOS (Median, IQR)	8 (10)	9 (10)	15 (17)	*<0.01*

WBCs, white blood cells; CRP, C-reactive protein; LOS, length of stay.

**Table 2:** Demographic and clinical characteristics of patients admitted prior to and during the COVID-19 pandemic in 2019 and 2020 (*n* = 640).

Patient Characteristics			Duration of Antibiotic Use			*p*-Value
			≤5 Days *n* = 463	6–7 Days *n* = 109	>8 Days *n* = 68	
			*n (%)*	*n (%)*	*n (%)*	
Demographic characteristics	Year	2019	237 (51.2)	53 (48.6)	32 (47.1)	0.3
		2020	226 (48.8)	56 (51.4)	36 (52.9)	
	Gender	Male	227 (49)	60 (55)	34 (50)	0.6
		Female	236 (51)	49 (45)	34 (50)	
	Patient Outcomes	Discharged	384 (82.9)	97 (89)	61 (89.7)	0.9
		Died	79 (17.1)	12 (11)	7 (10.3)	
	Allergy		36 (7.8)	11 (10.1)	6 (8.82)	0.6
Clinical characteristics	Indication	CAP	199 (43)	38 (34.9)	25 (36.8)	0.9
		HAP	82 (17.7)	21 (19.3)	16 (23.5)	0.7
		VAP	4 (0.9)	1 (0.9)	1 (1.5)	-
		COPD infective exacerbation	32 (6.9)	9 (8.3)	3 (4.4)	0.6
		COVID-19 pneumonia	35 (7.6)	8 (7.3)	4 (5.9)	0.6
		Unspecified	111 (24)	32 (29.4)	19 (27.9)	0.4
	Comorbidities	Hypertension (HPN)	212 (45.8)	47 (43.1)	32 (47)	0.5
		Heart failure (HF)	63 (13.6)	24 (22)	8 (11.8)	0.3
		Hypercholesterolemia	69 (14.9)	17 (15.6)	12 (17.6)	0.3
		Diabetes mellitus (DM)	79 (17.1)	22 (20.1)	18 (26.5)	0.6
		Asthma	41 (8.9)	11 (10.1)	4 (5.9)	0.1
	Chest X-rays	Pneumonia	66 (14.3)	16 (14.5)	11 (16.2)	0.3
		No pneumonia	107 (23.1)	22 (20.1)	18 (26.5)	

CAP, community-acquired pneumonia; HAP, hospital-acquired pneumonia; VAP, ventilator-associated pneumonia; COPD, chronic obstructive pulmonary disease; COVID-19, coronavirus; HPN, hypertension; HF, heart failure; and DM, diabetes mellitus. The *p*-value is significant if less than 0.5.

**Table 3:** Most frequently used antibiotics for patients with respiratory tract infections in 2019 and 2020.

Antibiotic	Duration Category			*p*-Value
	≤5 Days *n* = 463	6–7 Days *n* = 109	>8 Days *n* = 68	
	*n* (%)	*n* (%)	*n* (%)	
Amoxicillin	16 (3.5)	4 (3.7)	1 (1.5)	-
Amoxicillin/Clavulanic Acid	283 (61.1)	61 (56)	36 (52.9)	-
Azithromycin	5 (1.1)	0 (0)	2 (2.9)	-
Benzylpenicillin	3 (0.6)	1 (0.9)	1 (1.5)	-
Ceftazidime	6 (1.3)	1 (0.9)	2 (2.9)	-
Ciprofloxacin	11 (2.4)	2 (1.8)	4 (5.9)	-
Clarithromycin	23 (5)	7 (6.4)	2 (2.9)	-
Levofloxacin	50 (10.8)	6 (5.5)	3 (4.4)	-
Metronidazole	4 (0.9)	1 (0.9)	4 (5.9)	*0.01*
Piperacillin/Tazobactam	53 (11.4)	22 (20.2)	12 (17.6)	*0.007*

**Table 4:** Characteristics between short- and long-antibiotic-course treatment groups and appropriateness of antibiotics. Significant *p*-value is <0.05.

	Indication (*n*, %)	Duration of Antibiotic Use			*p*-Value
		≤5 Days *n* = 463	6–7 Days *n* = 109	>8 Days *n* = 68	
		*n (*%)	*n (*%)	*n (*%)	
Appropriateness of antibiotics	CAP (262, 40.9%)	84 (18.1)	25 (22.9)	14 (20.6)	*0.02*
	HAP (119, 18.6%)	45 (9.7)	11 (10.1)	7 (10.3)	0.7
	VAP (6, 0.9%)	3 (0.6)	1 (0.9)	1 (1.5)	0.6
	COPD infective exacerbation (44, 6.9%)	17 (3.7)	5 (4.6)	1 (1.5)	0.6
	COVID-19 pneumonia (47, 7.3%)	8 (1.7)	1 (0.9)	1 (1.5)	0.4
	Unspecified (162, 25.3%)	7 (1.5)	2 (1.8)	0 (0)	*0.07*
	Overall (640, 100%)	164 (35.4)	45 (41.3)	24 (35.3)	0.5

CAP, community-acquired pneumonia; HAP, hospital-acquired pneumonia; VAP, ventilator-associated pneumonia; COPD, chronic obstructive pulmonary disease; COVID-19, coronavirus.

## Data Availability

The datasets presented in this article are not readily available, according to the institution’s policy. Requests to access the datasets should be directed to r.a.elshenawy@herts.ac.uk.
